# Soil Nitrate Monitoring for Turfgrass Sod Farms and Other Turf Areas

**DOI:** 10.1100/tsw.2001.303

**Published:** 2001-11-02

**Authors:** W. Michael Sullivan, Zhongchun Jiang

**Affiliations:** Department of Plant Sciences, University of Rhode Island, Kingston 02881, USA

**Keywords:** golf course, ion exchange resin capsule, nitrate flux, nitrate leaching, nutrient management, nutrient monitoring, sod farm, soil, soil nutrient flux, turfgrass, turfgrass management

## Abstract

Studies with established turf and golf courses have indicated minimal risk of nitrate pollution of groundwater resulting from turfgrass management, but soil nitrate flux in turfgrass sod production farms and golf courses has received less attention. Information about nitrate-N flux at a particular location can be helpful to the sod producer or the golf course manager when efficiently applying N fertilizers and minimizing risk of nitrate pollution. We used an ion exchange resin capsule system to continuously monitor soil nitrate-N fluxes at 12 sites in southern Rhode Island, including turfgrass sod production farms and a low-maintenance environment. Four capsules were placed in the soil at each site and retrieved at intervals coinciding with management and meteorological events to determine nitrate ion accumulation. We found that the golf course green exhibited significantly higher nitrate-N fluxes than the sod farms and the low-maintenance turf. There was significant interaction between sampling date and study site, indicating that seasonal variation in soil nitrate-N fluxes was affected by turfgrass management. The cultural practice of late fall fertilization to stimulate early spring growth in the following year appeared to present some risk of nitrate loss during the winter from the golf course greens in our region. We conclude that site-specific and time-relevant monitoring is needed to produce and manage turfgrasses in an environmentally sound manner.

## INTRODUCTION

Nitrate leaching resulting from improper fertilizer use in agricultural production and other landscape settings has been reported as a significant nonpoint source of environmental pollution and is considered a major contributor to the formation of a vast hypoxic region in the Gulf of Mexico[1,2]. According to the 1997 Census of Agriculture of the U.S. Department of Agriculture[3], expenditures on commercial fertilizer in the U.S. were $9.6 billion in 1997, and the chemical fertilizer was applied to 233.5 million acres. Fertilizer use data from the Rhode Island Department of Environmental Management (RIDEM), Division of Agriculture[4], shows that for the years July 1, 1987 through June 30, 1993, approximately 16,073 tons of fertilizer with approximately 2000 tons of nitrogen (N) were used in the state of Rhode Island. Farm fertilizer use accounted for 20%, while nonfarm use accounted for 80% of the total tonnage in this state.

Numerous studies on N movement in turfgrass–soil ecosystems have been conducted to optimize turfgrass management and minimize risk of environmental pollution. Petrovic[5] reviewed research papers published before 1990 and found that the turfgrass plants absorbed 5 to 74% of fertilizer N, while the soil stored 15 to 26%. The remaining portion of fertilizer N could be lost to the atmosphere (0 to 36%), to groundwater (10 to 53%), and to surface waters (0 to 7%). These proportions could change with turfgrass management practices, including fertilization rate and timing. Cohen et al.[6] reviewed 19 studies on a total of 40 golf courses, mostly in the eastern and western states of the U.S., and found that the golf courses studied did not cause widespread and/or repeated water quality impacts. Only 3.6% of the 849 water samples exceeded the 10 mg $l^{-1}$ maximum contaminant limit for nitrate-N and were attributed to prior agricultural land use.

In the state of Rhode Island, turfgrass sod production is a significant agricultural sector. An acreage equivalent to approximately 7.8% of the state's crop land is used for turfgrass sod production, with sod total sales reaching 31.2% of the state's all-crop outputs in 1998. The majority of sod farms are sited over aquifers with highly permeable sandy and loamy soils, and potential nitrate loss during production and immediately after sod harvest is a concern. In a study by Jiang et al.[7], nitrate loss within an established turf-soil ecosystem was investigated following the sudden death of the turf. The study indicated that nitrate concentration in the water collected at a soil depth of 60 cm below killed turf was elevated beyond that of healthy turf within 2 months following turf death. Leachate reached a high of 38 mg nitrate-N $l^{-1}$. Significant differences in KCl-extractable nitrate were also observed, with levels in killed turf being two to four times higher than in healthy turf. A study by Sullivan and Sawyer[8] found that N losses from sod farms could be over 56 kg/ha when irrigation was not scheduled properly, compared to less than 11.2 kg/ha when irrigation was scheduled properly. Other studies with established turf systems in Rhode Island have demonstrated dramatic seasonal fluctuations in nitrate concentration in soil water collected under turf areas maintained with various management strategies[9,10,11,12], and great potentials of modifying management practices for reducing N losses from turf[13,14,15]. Comparable studies have not been conducted extensively on sod farms and golf courses, which are built in some exceptionally beautiful and environmentally sensitive areas.

The research reported here was to assess soil nutrient fluxes associated with sod production and golf course management in the state of Rhode Island. Our objective was to monitor nitrate-N fluxes at the level of the farm and the golf course green. Through continuous, multiyear monitoring at multiple sites, including both sod farms and golf courses, using an ion exchange resin capsule system, we expected to gain further information about the impact of overall turfgrass management in sod production and golf course maintenance as well as the effect of seasonality on the fluxes and potential losses of soil nutrients, including nitrate. This resin capsule system and the information obtained with it can be helpful to farmers, golf course superintendents, and other turf managers, in applying fertilizers at the right times of the year and avoiding nutrient loss from unnecessary or ill-timed applications, thus preventing nitrate pollution of ground- and surface waters.

## METHODS

An ion exchange resin capsule system was used for monitoring soil nutrient levels. This system provides a simple, convenient, and repeatable methodology[16,17,18]. The resin capsule is sensitive to soil environmental factors that regulate the availability of nutrient elements to plants and allows simultaneous, independent accumulation of cations and anions. Similar techniques involving exchange resins have been used for monitoring soil nutrient availability[19,20]. The ion exchange resin capsules that were used in this study (type PST-1, UNIBEST, Inc., 3535 Stucky Rd., Bozeman, MT) were factory prepared and easy to use. The PST-1 capsules are spherical resin balls encapsulated in a mesh plastic fabric with a diameter of approximately 2 cm and weight of less than 10 g.

During sampling, a capsule was placed in a socket on the bottom end (Fig. 1A) of PVC tubing (1.25 cm in diameter) inside an access tube (3.25 cm in diameter) and secured to the bottom end of the tubing with a compression ring retainer (Fig. 1B). A screw cap with a spring against the top end of the tubing inside the access tube was placed on the top end of the access tube, and when the screw cap was tightened, it would push the tubing downward. The access tube carrying the tubing and capsule was previously inserted into the soil through a valve box wall (Fig. 1C), and when the access screw cap was tightened, the spring pushed the capsule into contact with the soil. At each monitoring site, four access tubes were installed at soil depths of approximately 20 cm. A cover was placed on top of the valve box (Fig. 1D) so that the monitoring system would not interfere with normal operations of turfgrass management. Longer access tubes (not shown), approximately 3 m in length, were used at the golf course green.

A total of 12 monitoring sites, located at sod production farms, a low-maintenance turf, and a golf course green, were selected based on the turfgrass species, management practices, and location considerations. The farms and the golf course names will not be mentioned in this paper, and a code assigned to each will be used to avoid disclosure. Turfgrass species and soil infiltration rates, along with the monitoring dates and the number of samples taken, are presented in Table 1. Production cycles prevented installation of the monitoring systems at all sites at the same chronological time, and sod harvest determined duration of monitoring at each site. However, once the monitoring system was installed at a site, we added the site to a uniform schedule for sampling and replacing capsules throughout the study. This project is ongoing, and more sites are being considered. Selected sites and turfgrass species used are typical of sod farms in southern New England in terms of management intensity and diversity. Two basic sod products were being produced, one consisting of a mixture of predominantly (85 ± 5%) Kentucky bluegrass (*Poa pratensis* L.) and a small amount (15 ± 5%) of fine fescues (*Festuca* spp.), and the other of exclusively creeping bentgrass (*Agrostis palustris* Huds.).

Handling of capsules and desorption of accumulated nitrate ions from the retrieved capsules followed the protocol of the capsule manufacturer, as provided on the World Wide Web (http://www.unibestinc.com). Briefly, the capsule was placed in a container with a 50-ml capacity, and 20 ml of 2 *N* HCl was added to the container. The container was capped and shaken on a reciprocating shaker for 20 min. The extracted solution was poured into a bottle with a 60-ml capacity, and another 20 ml of 2 *N* HCl was added to the container. A total of three extraction cycles were completed, and all extracts were combined into the same bottle. After a sufficient volume of a concentrated NaOH solution was added to neutralize the extract solution, it was analyzed for nitrate concentration, using a rapid flow analytical system (RFA 300, Alpkem, Oregon).

The formula for calculating nitrate-N flux is given by:

$$\text{flux} = (cV/A)/d \qquad (1)$$

or

$$\text{flux} = (cV/A)/P \qquad (2)$$

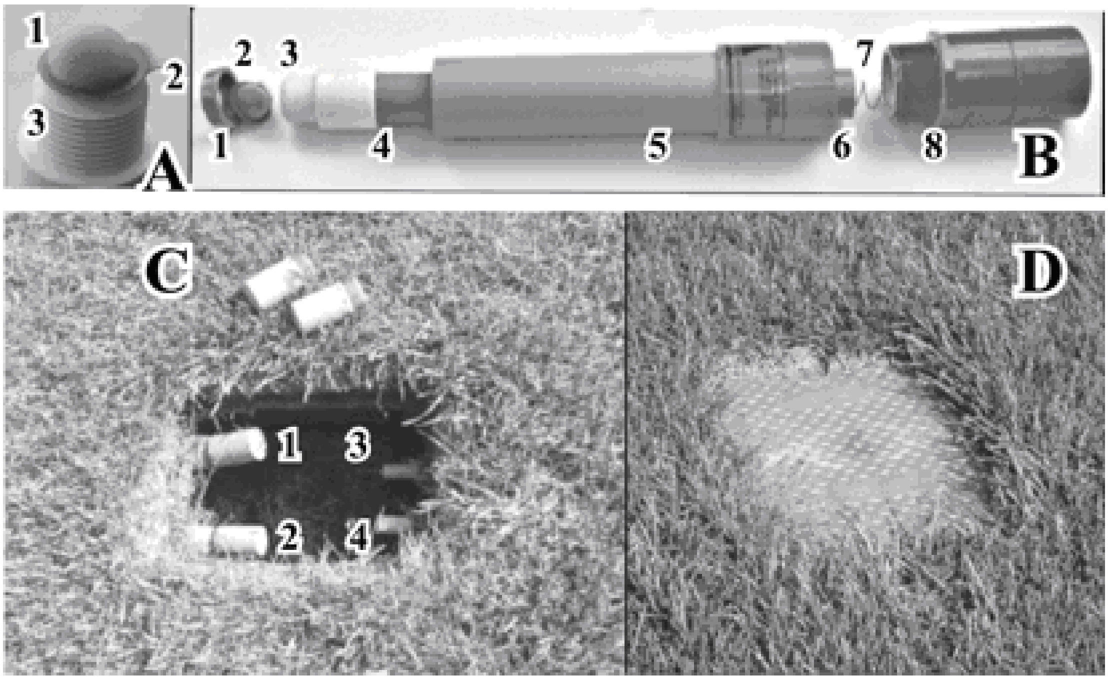

**FIGURE 1.** Ion exchange resin capsule system for monitoring soil nutrient fluxes at sod farms and golf courses. **(A)** A resin capsule (1) with a flange (2) is placed in the socket (3) of a carrier assembly. **(B)** A compression ring (1) is used to secure the capsule (2) to the socket (3) at the lower end of a 1.25-cm diameter PVC tubing assembly (4) inside a 3.25-cm diameter access tube (5). The upper end (6) of the assembly is attached to a spring (7) secured to a screw cap (8). When the screw cap is turned on, it pushes the assembly down so that the capsule comes into close contact with soil. **(C)** Four access tubes are seen in an installation box. Each of the tubes carrying a capsule is inserted into a soil pit through a hole on a side wall of the installation box, two tubes (1, 2) on the left and two (3, 4) on the right, and two long tubes (1, 4) reaching a deeper soil depth and two short tubes (2, 3) reaching a shallower depth. **(D)** The installation box is covered to avoid interfering with normal operations. The box is excavated at the time of sod harvest.

**TABLE 1**
**Turfgrass Species, Period Monitored, Soil Infiltration Rate, and Sample Number Taken from 12 Monitoring Sites in Southern Rhode Island**

| Site[a] | Turfgrass | Period Monitored | Infiltration (mm $h^{-1}$) | No. of Samples |
|---|---|---|---|---|
| 1 | Kentucky bluegrass + Fine fescue | January 2000 – present[b] | 50.8 | 52 |
| 2 | Kentucky bluegrass + Fine fescue | January 2000 – August 2000 | 50.8 | 28 |
| 3 | Creeping bentgrass | January 2000 – October 2000 | 63.5 | 36 |
| 4 | Creeping bentgrass | January 2000 – present | 38.1 | 52 |
| 5 | Creeping bentgrass | January 2000 – present | 38.1 | 52 |
| 6 | Kentucky bluegrass + Fine fescue + Perennial ryegrass | January 2000 – present | — | 52 |
| 7 | Kentucky bluegrass + Fine fescue | January 2000 – present | 96.5 | 48 |
| 8 | Kentucky bluegrass + Fine fescue | January 2000 – September 2000 | 88.9 | 28 |
| 9 | Creeping bentgrass | January 2000 – October 2000 | 38.1 | 36 |
| 10 | Creeping bentgrass | August 2000 – present | — | 28 |
| 11 | Kentucky bluegrass + Fine fescue | August 2000 – present | — | 20 |
| 12 | Creeping bentgrass | August 2000 – present | — | 28 |

[a] Site 6 was a low-maintenance turf, and site 12 was a golf course green. Other sites were in production of turfgrass sod.
[b] As of May 2001, monitoring was continuing.

where c is the concentration of nitrate-N in the final neutralized HCl extract obtained from the retrieved capsule (mg $l^{-1}$), V is the volume of the final neutralized extract (ml), A is the hemispherical surface of the capsule ($cm^2$), d is the number of days when the capsule was in the soil, and P is precipitation (mm) for the period monitored. The value was converted to pounds of nitrate-N per 1000 square feet per day or per inch of precipitation, for the purpose of comparing with published results and making recommendations regarding fertilizer application to producers and maintenance staff. In this paper, values are reported in g N $m^{-2}$ $d^{-1}$ and g N $m^{-2}$ $mm^{-1}$.

Statistical analysis was performed with the SAS Systems for Windows, Version 8 (SAS Institute, Inc., Cary, NC). The GLM (General Linear Model) procedure was used for analysis of variance, and least significant difference test was used to separate significant means.

## RESULTS AND DISCUSSION

A total of 460 resin capsules were retrieved and analyzed during the period between January 2000 and May 2001. The grand mean of nitrate-N flux was 0.01 g N $m^{-2}$ $d^{-1}$ or 0.002 g N $m^{-2}$ $mm^{-1}$ precipitation. Because our data set was unbalanced, i.e., some sites monitored earlier and some later, we used GLM procedure to analyze the overall variance (Tables 2 and 3). The results indicated that sampling date and study site significantly ($p < 0.0001$) affected nitrate-N fluxes in the soil expressed as g N $m^{-2}$ $d^{-1}$ (Table 2) or g N $m^{-2}$ $mm^{-1}$ precipitation (Table 3). We also observed significant interaction between date and site, indicating variation in the seasonal nitrate-N fluxes among the sod farms, the low-maintenance turf site, and the golf course green. The soil depth at which resin capsules were placed was determined to be approximately the same and thus did not result in a significant difference in nitrate-N fluxes (Tables 2 and 3). Therefore, we used the mean of all four samples from a capsule installation box to represent the site on each date.

Although the interaction between sampling date and study site necessitated separate analyses of the data for each date, one of our objectives was to assess site differences in the overall mean of the nitrate-N flux (Fig. 2) and to compare influences of different management intensities on the nitrate-N flux. All the study sites, except sites 5, 11, and 12, had soil nitrate-N fluxes of less than 0.01 g N $m^{-2}$ $d^{-1}$. Site 12 was a golf course green with a sandy soil mix, creeping bentgrass, and the aggressive maintenance required of golf course environments: close mowing, frequent irrigation, and increased fertilization. This site had a significantly higher nitrate-N flux per day than any other site monitored. Site 5 was a sod farm producing creeping bentgrass sod on a very sandy soil, and site 11 was a newly seeded sod field using predominantly Kentucky bluegrass. Both sites had relatively higher nitrate-fluxes (Fig. 2), which could lead to nitrate leaching into groundwater when rainfall was heavy or irrigation excessive. Site 6 was maintained as a turf with minimum input, and it saw very little nitrate-N fluxes, which would effectively minimize nitrate leaching. Other studies[12] with long-established turfs in Rhode Island found that these turf-covered areas

**TABLE 2**
**Analysis of Variance Results Using GLM Procedure for Nitrate Flux Expressed as g N $m^{-2}$ $d^{-1}$**

| Source | DF | Sum of Squares | Mean Square | F | P |
|---|---|---|---|---|---|
| Model | 115 | 0.11284099 | 0.00098123 | 3.80 | <0.0001 |
| Error | 344 | 0.08876148 | 0.00025803 | | |
| Date | 12 | 0.01402949 | 0.00116912 | 4.53 | <0.0001 |
| Site | 11 | 0.04383021 | 0.00398456 | 15.44 | <0.0001 |
| Soil depth | 1 | 0.00003825 | 0.00003825 | 0.15 | 0.7005 |
| Date × Site | 91 | 0.05494304 | 0.00060377 | 2.34 | <0.0001 |

**TABLE 3**
**Analysis of Variance Results Using GLM Procedure for Nitrate Flux Expressed as g N $m^{-2}$ $mm^{-1}$ of Precipitation**

| Source | DF | Sum of Squares | Mean Square | F | P |
|---|---|---|---|---|---|
| Model | 115 | 0.00658257 | 0.00005724 | 3.85 | <0.0001 |
| Error | 344 | 0.00511820 | 0.00001488 | | |
| Date | 12 | 0.00078967 | 0.00006581 | 4.42 | <0.0001 |
| Site | 11 | 0.00287031 | 0.00026094 | 17.54 | <0.0001 |
| Soil depth | 1 | 0.00000000 | 0.00000000 | 0.00 | 0.9918 |
| Date × Site | 91 | 0.00292259 | 0.00003212 | 2.16 | <0.0001 |

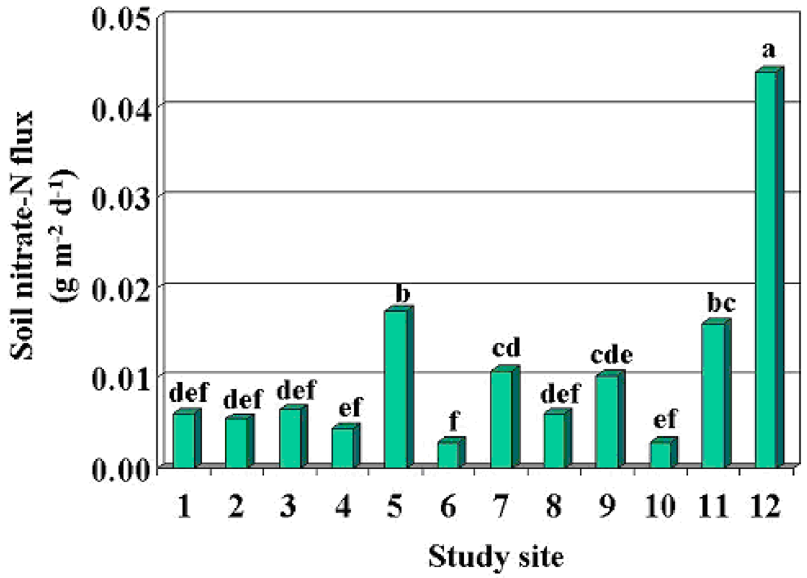

**FIGURE 2.** Means of soil nitrate-N fluxes on a per-day basis at 12 study sites. Bars labeled with the same letters are not significantly different based on least significant difference test at $p < 0.05$.

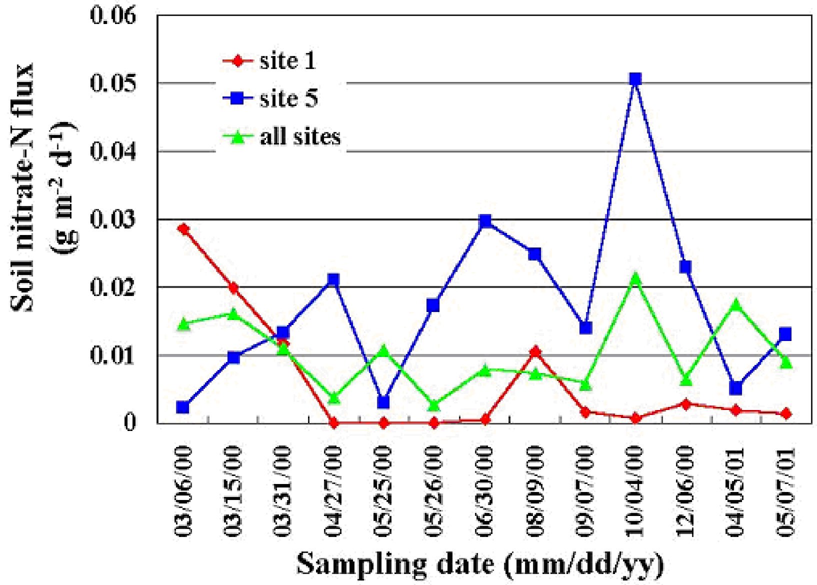

**FIGURE 3.** Seasonal variation in soil nitrate-N fluxes on a per-day basis at site 1 and site 5. The mean of all sites for each sampling date is also presented for comparison. For site 1 and site 5, each data point is the mean of four samples. Standard deviation bar at each data point was omitted to avoid congestion. Least significant differences for comparing values between dates were 0.01 g N $m^{-2}$ $d^{-1}$ for site 1, 0.02 for site 5, and 0.005 for the means of all sites.

had a very low potential of nitrate leaching when no more than 15 g N $m^{-2}$ $d^{-1}$ was applied annually. When a dead turf was reseeded, the initial establishment period resulted in increased nitrate concentrations in the soil water, but soon thereafter, the nitrate level in the reseeded area decreased[21]. The high nitrate-N flux in the soil of the newly established sod (Fig. 2, site 11) and the low nitrate flux in the soil of the low-maintenance turf (Fig. 2, site 6) in the present study were in agreement with these reports.

To illustrate the magnitude of seasonal variation in soil nitrate-N fluxes, we presented data of site 1 and site 5 as two examples, along with the mean of all sites monitored for each date (Fig. 3). In addition to the fact that these two sites were monitored throughout the period reported, these two sites also differed in turfgrass species used and measured infiltration rate, site 1 being somewhat higher than site 5 (Table 1). At site 1, the nitrate-N flux on a per-day basis decreased during the early spring (March 6, 2000 through April 27, 2000) and remained undetectable or very low during the remaining seasons, except an early fall peak (August 9, 2000, Fig. 3), which coincided with an early fall fertilization. An earlier study in a nearby area found a similar seasonal variation pattern in nitrate concentration of the soil water collected at 60-cm depth[11]. In contrast, site 5 used creeping bentgrass and required intensive management, including 0.3-cm mowing height, and exhibited a very different pattern of seasonal variation (Fig. 3). Nitrate-N flux at site 5 increased steadily in March and April 2000. A decrease in June (May 25, 2000 through June 26, 2000) and another in early fall (August 9, 2000 through September 7, 2000) were observed. As a result of a late fall N application, the nitrate-N flux at this site jumped to the highest point on October 4, 2000, and then decreased steadily through the winter and into the spring of 2001. Late fall N application to stimulate early growth in the following spring has been a widely used practice in the cool regions. Our results appeared to indicate that, with creeping bentgrass, this practice creates additional risk of nitrate leaching in the winter, when precipitation is heavy in our region.

Because precipitation presumably facilitates nitrate leaching when the soil nitrate flux is high, we analyzed the nitrate-N flux data on a precipitation basis and found similar site differences (Fig. 4) and similar seasonal variations in the nitrate-N flux per millimeter of precipitation (Fig. 5) as with the flux on a per-day basis. Fig. 4 shows that most of the sites monitored had soil nitrate-N fluxes of less than 0.002 g N $m^{-2}$ $mm^{-1}$ of

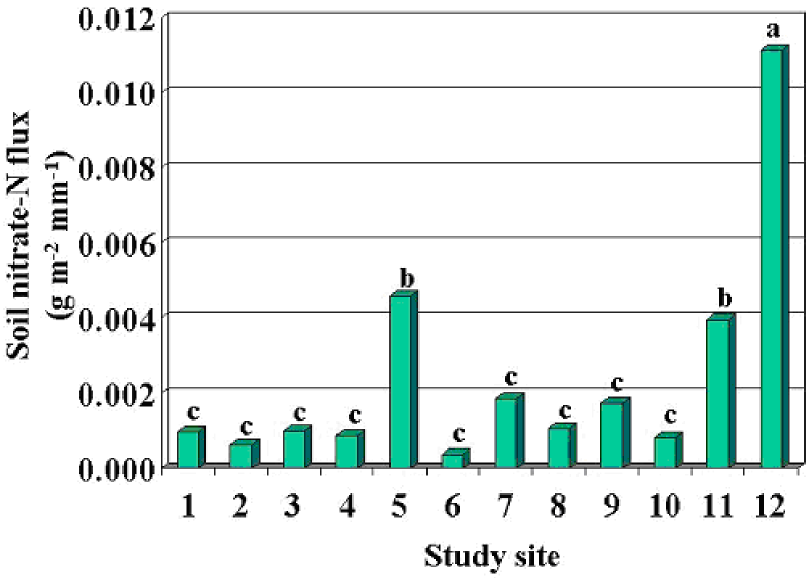

**FIGURE 4.** Means of soil nitrate-N fluxes based on a millimeter of precipitation at 12 study sites. Bars labeled with the same letters are not significantly different based on least significant difference test at $p < 0.05$.

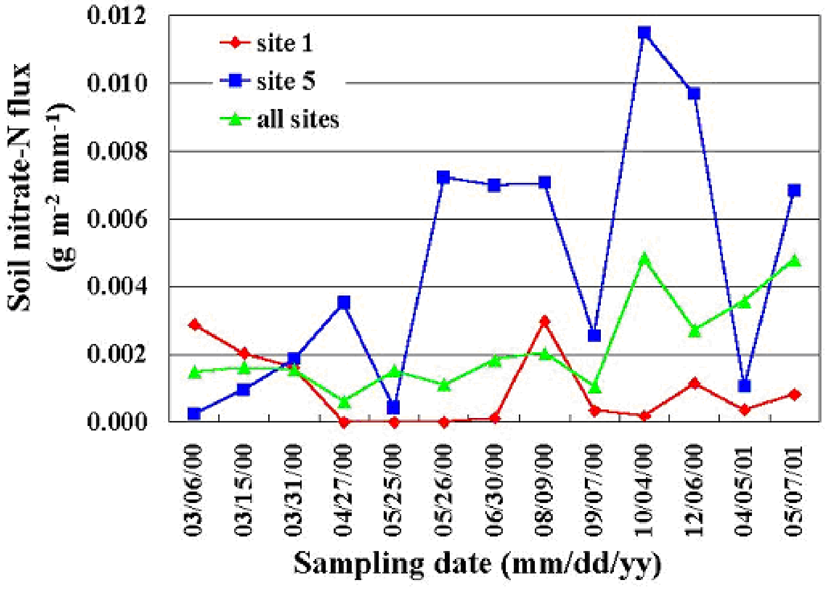

**FIGURE 5.** Seasonal variation in soil nitrate-N fluxes based on a millimeter of precipitation at site 1 and site 5. The mean of all sites for each sampling date is also presented for comparison. For site 1 and site 5, each data point is the mean of four samples. Standard deviation bar at each data point was omitted to avoid congestion. Least significant differences for comparing values between dates were 0.004 g N $m^{-2}$ $mm^{-1}$ of precipitation for site 1, 0.008 for site 5, and 0.002 for the means of all sites.

precipitation. However, 1 mm of precipitation resulted in a flux density of more than 0.01 g N $m^{-2}$ in the golf course green, whereas the same amount of precipitation resulted in significantly lower flux densities, far less than 0.001 g N $m^{-2}$, in a typical low-maintenance turf (Fig. 4, site 6). The golf course green consistently exhibited higher nitrate-N fluxes than the average site throughout the summer of 2000 and late fall through winter of 2001. Again, our results indicated that management intensities associated with the particular type of turfgrass and soil could significantly impact the potential of nitrate leaching, and that irrigation and fertilization must be carefully timed to minimize nitrate leaching under such management.

## CONCLUSIONS

We used an ion exchange resin capsule system to continuously monitor a dozen sites at sod farms and a golf course in the state of Rhode Island and found significant differences in the soil nitrate flux among the sites. Our results suggested that management practices and intensities associated with the turfgrass species and soil type seen at those sites may have a profound impact on the potential for nitrate leaching. Seasonal variation in the soil nitrate flux appeared to be influenced by the soil characteristics, turfgrass species, and management practices unique to the individual sites monitored. We conclude that site-specific and time-relevant monitoring is needed to produce and manage turfgrasses in a more environmentally friendly manner.

## ACKNOWLEDGMENTS

We would like to thank John W. Pote for his work with installing and retrieving resin capsules and laboratory analysis and Carl D. Sawyer for providing weather data for this project. The U.S. Environmental Protection Agency — New England Region provided funding for this project, and we acknowledge the support with appreciation. We especially thank our unnamed turfgrass producers and managers for their cooperation.

| | | |
|---|---|---|
| Received: | July | 16, 2001 |
| Revised: | October | 8, 2001 |
| Accepted: | October | 15, 2001 |
| Published: | November | 2, 2001 |

## BIOSKETCH

**Zhongchun Jiang, Ph.D.,** is Assistant Research Professor, Plant Sciences, University of Rhode Island. Research interests include nutrient management, plant physiology, and molecular biology of turfgrasses and forage grasses. Current effort is focused on soil nutrient monitoring and management for Rhode Island sod farms and golf courses, nitrate uptake efficiency and nitrate assimilation rate, the molecular aspects of nitrate transporters, and nitrate reductases of Kentucky bluegrass.
